# Neurotoxic syndrome induced by clomipramine plus risperidone in a patient with autistic spectrum disorder: serotonin or neuroleptic malignant syndrome?

**DOI:** 10.1186/s12991-015-0073-z

**Published:** 2015-11-14

**Authors:** Kalliopi N. Nikolaou, Rossetos Gournellis, Ioannis Michopoulos, Georgios Dervenoulas, Christos Christodoulou, Athanasios Douzenis

**Affiliations:** Second Department of Psychiatry, Medical School, National and Kapodistrian University of Athens, University General Hospital “Attikon”, 1 Rimini Street, GR-124 62 Athens, Greece; Second Department of Neurology, Medical School, National and Kapodistrian University of Athens, University General Hospital “Attikon”, 1 Rimini Street, GR-124 62 Athens, Greece

**Keywords:** Neuroleptic malignant syndrome, Serotonin syndrome, Risperidone, Clomipramine, Autism

## Abstract

To the best of our knowledge, there are no case studies of serotonin syndrome (SS) in patients with autism spectrum disorder. We report the case of a 33-year-old male who presented SS under the combined use of clomipramine and risperidone. More specifically, within 2 days after clomipramine (10 mg/BID-two times a day) was added to risperidone (4 mg/OD-once a day), mirtazapine 45 mg/OD and alprazolam (0,5 mg/TID-three times a day) he began to present mental, neurological and autonomic symptoms. All his psychopathological manifestations and laboratory findings normalized after the above-mentioned drugs’ discontinuation, and the administration of supportive medical care and lorazepam 2,5 mg/TID. The diagnosis of serotonin syndrome was challenging due to the relatively low dose of clomipramine, an increase of risperidone which had taken place before clomipramine administration and clinical symptoms which could be attributed to both serotonin and neuroleptic malignant syndrome.

## Background

Autism spectrum disorder (ASD) is a complex neurodevelopmental disorder characterized by deficits in verbal and non-verbal communication and social functioning, restricted, repetitive or stereotypical patterns of interest and/or behaviour since the early developmental period. It is considered to constitute a continuum of heterogeneous life-long disorders, three or four times more prevalent in males than in females. [1].

Serotonin syndrome (SS) is characterized by cognitive and behavioural (e.g. confusion, agitation), autonomic (e.g. fever, diaphoresis, tachycardia) and neurological (e.g. myoclonus, rigidity) symptoms [2] and is a manifestation of overactivation of peripheral and central 5-HT1A or 5-HT2A receptors [3]. Neuroleptic malignant syndrome (NMS) is characterized by mental state changes, bradykinesia, rigidity, autonomic dysfunction and hyperthermia, and it is suggested to be caused by dopamine D2 receptor blockade [4, 5]. Both syndromes are uncommon, though life-threatening. However, SS is caused by increased intra-synaptic 5-HT levels, a condition of toxicity in which everyone is liable, whereas NMS is an idiosyncratic response.

Clomipramine blocks serotonin and also noradrenaline reuptake presumably increasing thus their neurotransmission. It has been used in adult patients with ASD [6, 7] with limited and conflicting evidence of effect, though well tolerated [8]. Furthermore, there are only scarce reports of risperidone—an atypical antipsychotic blocking dopamine D2 and serotonin 5HT2A receptors—in ASD patients which is usually administrated in doses ranging from 1 to 4 mg/day [9–11].

Although dopaminergic and serotonergic neurotransmission systems have been shown to be dysfunctional in autism [12, 13], to the best of our knowledge, no cases of SS have been reported in patients with ASD, whereas cases of NMS are very rare [14].

In the following, we report a patient with ASD who presented SS under the combined use of clomipramine and risperidone, mirtazapine and alprazolam and the diagnosis of SS was challenging due to the remarkable overlap between symptoms of both neurotoxic syndromes.

## Case presentation

A 33-year-old male with ASD, without accompanying intellectual and language impairment and disorder severity level 1-requiring support [1] was admitted to our Department with psychomotor agitation, muscular rigidity (neck, upper and lower extremities), cogwheel rigidity, sweating, pallor, tremor, hyperreflexia, clonus, delayed responsiveness, insomnia, oversalivation, bradykinesia and akathisia. The patient was extremely anxious with depressive mood, well orientated and did not have psychotic symptoms. He had a body temperature of 37.9 °C, tachycardia of 129 beats/min and marginally elevated blood pressure (140/83 mmHg). Laboratory examination revealed elevated serum CPK: 3220 IU/L. Urine analysis and radiological investigations, including cranial computed tomography (CT), chest X-ray and abdominal sonography, were unremarkable. Thorough laboratory and clinical work-up did not reveal any signs of systemic infection.

Since his childhood, the patient had a long history of marked difficulties in peer, social and emotional interaction, in developing and maintaining relationships, and suffered from extreme distress when exposed even to small life changes. At the age of eight, he was diagnosed as suffering from “Asperger syndrome with IQ within the normal range”. He was always viewed as eccentric with no sexual affairs. He studied journalism in a private college and he worked (in e-shops) only under the supervision from his father.

At the age of 18, he experienced a major depressive episode treated successfully with mirtazapine (30 mg/OD-once a day). Since then, mirtazapine was administrated constantly at the same dose. Occasionally, risperidone was added at a low dose, no more than 2 mg/day and for about 1 month each time. Over the past month, he was unable to adapt to his working environment and had started feeling depressed and anxious about his physical and mental health. Then, alprazolam was added (0,5 mg/TID-three times a day), the dose of mirtazapine was increased to 45 mg/OD and risperidone was added gradually raised up to 4 mg/OD, within 2 weeks. Then and since the patient had no improvement, venlafaxine was started at the dose of 75 mg/OD but discontinued immediately after the first dosage due to the patient’s complaints of hand tremor and restlessness. One week later, after the patient having referred suicidal thoughts, clomipramine was added at the dose of 10 mg/BID-twice a day. Within 48 h, oversalivation, diaphoresis, anxiety, muscular and neck stiffness, akathisia, dysphagia in liquids, fever (38.0 °C) ameliorated by paracetamol and gastrointestinal complaints had developed. Risperidone and clomipramine were discontinued and the patient was admitted to our Department. At the day of admission, he was taking alprazolam (0,5 mg/TID) and mirtazapine (45 mg/OD), both immediately discontinued and supportive medical care as well as lorazepam 2,5 mg/TID was initiated. Within the following 10 days, muscular symptoms improved and CPK levels decreased to 83 IU/L (normal range 39–308). The patient’s psychopathological state markedly improved, all laboratory parameters normalized and he was finally discharged 4 weeks after admission.

## Conclusions

The differential diagnosis of our case includes several alternatives: our patient fulfilled six of the Sternbach’s [15] SS criteria namely agitation, myoclonus, hyperreflexia, sweating, tremor, fever, and nine of Radomski’s [16], in addition to the above insomnia, akathisia, and tachycardia. However, the exclusion criterion of “a recent change in dosage of a neuroleptic agent” [15, 16] was also fulfilled. Indeed, risperidone alone could have led to NMS due to its antagonistic activity at both serotonin and dopamine receptors. On the other hand, it had been administered to the patient several times in the past without producing such symptoms, nevertheless NMS is mainly an idiosyncratic rather than a dose-related phenomenon [17]. The addition of clomipramine, which has a weak antagonistic effect at dopamine receptors and is a potent serotonin reuptake inhibitor, could have further contributed to dopamine hypoactivity [18]. The diagnosis of NMS could be possible given that our patient fulfilled three major Levenson’s criteria [19], namely fever, rigidity and elevated creatine kinase, and two minor namely tachycardia and sweating. He also fulfiled one essential (administration of a neuroleptic drug), one major (elevated CPK) and one minor (extrapyramidal symptoms) of Nierenberg’s NMS criteria [20].

Mirtazapine—an alpha 2 antagonist—which enhances central noradrenergic and serotonergic activity might have caused NMS or SS but the patient had tolerated it well for almost 15 years. Furthermore, fever, tachycardia and his marginally elevated blood pressure could have been attributed to the anticholinergic adverse effects of both risperidone and clomipramine.

To put more emphasis on the distinction between the two syndromes, SS follows exposure to serotonin agonists, develops within 24 h and resolves rapidly with treatment, as well as its main symptoms are altered mental status, muscle rigidity, hyperreflexia, clonus, hyperkinesia, increased bowel sounds, and diaphoresis. On the other hand, NMS follows exposure to dopamine antagonists or rapid increase in dosage, develops more slowly within days or weeks and resolves with treatment in more than a week time, and it is characterized by cog-wheel or lead pipe rigidity, bradykinesia and extrapyramidal symptoms, encephalopathy, autonomic lability and hyperthermia [3, 4, 24]. Both SS and NMS share several common symptoms, besides it has been proposed that these syndromes may share similar pathophysiology [22–24]. Nevertheless, we consider as lending further support to SS diagnosis of our patient the relatively rapid escalation of symptoms within 24 h after clomipramine administration even in low dose, the presence of hyperreflexia, myoclonus and body temperature <380 C, as well as the lack of altered consciousness, severe autonomic and respiratory dysfunction, “lead pipe” rigidity and leukocytosis [2, 16, 20, 21].

Furthermore, patient’s 2D6 isoenzyme might have been overloaded producing thus a state of serotonin toxicity. More specifically, co-medication of risperidone might have increased the plasma concentration of clomipramine [25], whereas mirtazapine is not considered a potent inhibitor of 2D6 [26]. In addition, as Cano-Munoz et al. [27] have reported the co-administration of clomipramine with alprazolam might potentially produce SS possibly due to the latter’s mild serotoninergic action. Of note, drug interactions that may increase the risk of SS development are the co-administration of serotoninergic antidepressants (SSRIs, MAOIs, SNRIs, TCAs), opiates with antidepressants, l-tryptophan or buspirone with SSRIs, or the latter with olanzapine and/or risperidone. Consequently, in our case, the co-administration of clomipramine, which is among the most potent TCA serotonin reuptake inhibitors, alongside with mirtazapine, risperidone and alprazolam may have contributed to SS development [3, 28]. At this point, it should be noted that elevated serotonin levels have been observed in autism [29].

With regard to the patient’s treatment, it was consisted of discontinuation of serotoninergic agents and risperidone, supportive care and lorazepam administration. In SS cases treatment with benzodiazepines is well tolarated and established in mild to moderate cases. Benzodiazepines through their GABA-mimic action ameliorate patient’s agitation, mild hypertension, tachycardia and fever [2, 3, 28]. They are also considered as an advisable option in NMS cases. [22, 30].

Clinicians should be cautious about the development of SS in cases of clomipramine (even in low dose) and risperidone co-administration in patients with ASD.

## Consent

Written informed consent was obtained from the patient for publication of this case report and any accompanying images.

## References

[1] American Psychiatric Association (2013). Diagnostic and statistical manual of mental disorders.

[2] Birmes P, Coppin D, Schmitt L, Lauque D (2003). Serotonin syndrome: a brief review. CMAJ Can Med Assoc J.

[3] Volpi-Abadie J, Kaye AM, Kaye AD (2013). Serotonin syndrome. Ochsner J.

[4] Gillman P (2010). Neuroleptic malignant syndrome: mechanisms, interactions and causality. Mov Disord.

[5] Caroff SN, Mann SC, Campbell EC, Sullivan KA, Obeso J. Neuroleptic malignant syndrome. In: Frucht SJ, Fahn S, editors. Movement disorder emergencies: diagnosis and treatment. New Jersey: Humana Press Inc; 2005. p. 41–51.

[6] Brodkin ES, McDougle CJ, Naylor ST, Cohen DJ, Price LH (1997). Clomipramine in adults with pervasive developmental disorders: a prospective open-label investigation. J Child Adolesc Psychopharmacol..

[7] Remington G, Sloman L, Konstantareas M, Parker K, Gow R (2001). Clomipramine versus haloperidol in the treatment of autistic disorder: a double-blind, placebo-controlled, crossover study. J Clin Psychopharmacol.

[8] Hurwitz R, Blackmore R, Hazell P, Williams K, Woolfenden S. Tricyclic antidepressants for autism spectrum disorders (ASD) in children and adolescents. Cochrane Database Syst Rev. 2012;14(3). doi:10.1002/14651858.CD008372.pub2. 10.1002/14651858.CD008372.pub2 PMC12053881 22419332

[9] McDougle CJ, Holmes J (1998). A double-blind, placebo-controlled study of risperidone in adults with autistic disorder and other pervasive developmental disorders. Arch Gen Psychiatry.

[10] Raheja S, Libretto SE, Singh I (2002). Successful use of Risperidone in an adult with the pervasive developmental disorder, Asperger’s Syndrome: a case report. Br J Dev Disabil.

[11] Alexander RT, Michael DM, Gangadharan SK (2004). The use of risperidone in adults with Asperger syndrome. Int J Dev Disabil.

[12] Barthelemy C, Bruneau N, Cottet-Eymard JM, Domenech-Jouve J, Garreau B, Lelord (1988). Urinary free and conjugated catecholamines and metabolites in autistic children. J Autism Dev Disord.

[13] McDougle CJ, Scahill L, McCracken JT, Aman MG, Tierney E, Arnold LE (2000). Research units on paediatric psychopharmacology autism network: background and rationale for an initial controlled study of Risperidone. Child Adolesc Psychiatr Clin.

[14] Neuhut R, Lindenmayer J-P, Silva R (2009). Neuroleptic malignant syndrome in children and adolescents on atypical antipsychotic medication: a review. J Child Adolesc Psychopharmacol.

[15] Sternbach H (1991). The serotonin syndrome. Am J Psychiatry.

[16] Radomski JW, Dursun SM, Revely MA, Kutcher SP (2000). An exploratory approach to the serotonin syndrome; an update of clinical phenomenology and revised diagnostic criteria. Med Hypotheses.

[17] Whyte IM, Dart ERC (2004). Neuroleptic malignant syndrome. Medical toxicology.

[18] Haddow AM, Harris D, Wilson M, Logie H (2004). Clomipramine induced neuroleptic malignant syndrome and pyrexia of unknown origin. BMJ.

[19] Levenson JL (1985). Neuroleptic malignant syndrome. Am J Psychiatr.

[20] Nierenberg D, Disch M, Manheimer E, Patterson J, Ross J, Silvestri G, Surnmerhill E (1991). Facilitating prompt diagnosis and treatment of the neuroleptic malignant syndrome. Clin Pharmacol Ther.

[21] Isbister GK, Buckley NA (2005). Clomipramine and neuroleptic malignant syndrome. Literature on adverse reactions to psychotropic drugs continues to confuse. BMJ.

[22] Fink M (1996). Toxic serotonin syndrome or neuroleptic malignant syndrome?. Pharmacopsychiatry.

[23] Kontaxakis VP, Havaki-kontaxaki BJ, Christodoulou NG, Paplos KG, Christodoulou GN (2003). Olanzapine-associated neuroleptic malignant syndrome: is there an overlap with the serotonin syndrome?. Ann Gener Hosp Psychiatr.

[24] Steele D, Keltner NL, McGuiness TM (2011). Are neuroleptic malignant syndrome and serotonin syndrome the same syndrome?. Perspect Psychiatr Care.

[25] ANAFRANIL (clomipramine). http://www.novartis.com.au/pi_pdf/anf.pdf.

[26] REMERON (mirtazapine). http://www.fda.gov/ohrms/dockets/ac/04/briefing/2004-4065b1-26-tab11G-Remeron-Tabs-SLR016.pdf.

[27] Cano-Muñoz JL, Montejo-Iglesias ML, Yañez-Saez RM, Gálvez-Borrero IM (1995). Possible serotonin syndrome following the combined administration of clomipramine and alprazolam. J Clin Psychiatry.

[28] Gillman P (1999). The serotonin syndrome and its treatment. J Psychopharmacol.

[29] Anderson GM, Freedman DX, Cohen DJ, Volkmar FR, Hoder EL, McPhedran P (1987). Whole blood serotonin in autistic and normal subjects. J Child Psychol Psychiatry.

[30] Varelas P, Tamer Abdelhak T, Bhardwaj A, Mirski MA (2011). Neurolept*ic malignant sy*ndrome, malignant hyperthermia, and serotonin syndrome. Handbook of neurocritical care.

